# A Network Flow Approach to Predict Protein Targets and Flavonoid Backbones to Treat Respiratory Syncytial Virus Infection

**DOI:** 10.1155/2015/301635

**Published:** 2015-03-22

**Authors:** José Eduardo Vargas, Renato Puga, Joice de Faria Poloni, Luis Fernando Saraiva Macedo Timmers, Barbara Nery Porto, Osmar Norberto de Souza, Diego Bonatto, Paulo Márcio Condessa Pitrez, Renato Tetelbom Stein

**Affiliations:** ^1^Centro Infant, Pontifical Catholic University of Rio Grande do Sul (PUCRS), Avenue Ipiranga 6681, 90619-900 Porto Alegre, RS, Brazil; ^2^Clinical Research Center, Hospital Israelita Albert Einstein (HIAE), São Paulo, Brazil; ^3^Department of Molecular Biology and Biotechnology, Federal University of Rio Grande do Sul (UFRGS), 90619-900 Porto Alegre, RS, Brazil; ^4^Faculty of Informatics, Laboratory for Bioinformatics, Modelling & Simulation of Biosystems, Pontifical Catholic University of Rio Grande do Sul (PUCRS), 90619-900 Porto Alegre, RS, Brazil

## Abstract

*Background.* Respiratory syncytial virus (RSV) infection is the major cause of respiratory disease in lower respiratory tract in infants and young children. Attempts to develop effective vaccines or pharmacological treatments to inhibit RSV infection without undesired effects on human health have been unsuccessful. However, RSV infection has been reported to be affected by flavonoids. The mechanisms underlying viral inhibition induced by these compounds are largely unknown, making the development of new drugs difficult. *Methods.* To understand the mechanisms induced by flavonoids to inhibit RSV infection, a systems pharmacology-based study was performed using microarray data from primary culture of human bronchial cells infected by RSV, together with compound-proteomic interaction data available for *Homo sapiens*. *Results.* After an initial evaluation of 26 flavonoids, 5 compounds (resveratrol, quercetin, myricetin, apigenin, and tricetin) were identified through topological analysis of a major chemical-protein (CP) and protein-protein interacting (PPI) network. In a nonclustered form, these flavonoids regulate directly the activity of two protein bottlenecks involved in inflammation and apoptosis. *Conclusions.* Our findings may potentially help uncovering mechanisms of action of early RSV infection and provide chemical backbones and their protein targets in the difficult quest to develop new effective drugs.

## 1. Introduction

Respiratory syncytial virus (RSV) is a major cause of lower respiratory tract infection with high level of mortality in children around the world [1–3]. It is estimated that all children by two years of age have been infected by RSV and more than half of them are reinfected [4]. Moreover, RSV pathogenesis is notably associated with an increased airway resistance characterized as wheezing, diagnosed as bronchiolitis [2].

In the 1960 decade, a vaccine trial was performed with unexpected and tragic results [5]. Hence, effective preventive treatment to RSV infection is unavailable, since there is no vaccine against the virus. However, several prototypes are under study [6–9]. The prophylactic therapy with palivizumab, a humanized monoclonal antibody, has been shown to reduce the number of RSV hospitalizations in preterm infants [10], but the treatment has a very high cost, and it is administered only to children with risk factors for RSV bronchiolitis [11]. Another optional treatment against RVS infection is ribavirin. It is a nucleoside analog that introduces mutations into the RNA viral genome during replication and was previously used routinely for infants hospitalized with RSV. However, it has been associated with undesired side-effects and was not considered an effective treatment [12, 13].

The absence of a vaccine for RSV-induced bronchiolitis and the existence of few antiviral agents against RSV constitute very important problems in pediatric medicine. Thus, the development of novel anti-RSV drugs that can be administered orally or parenteral to children is extremely necessary.

A great variety of viruses have been reported to be inhibited by natural compounds, such as flavonoids [14–16]; however, the molecular mechanisms underlying such effects are largely unclear. In this sense, it is difficult to develop new drugs.

In a search to provide new insights for RSV treatments and to understand the multiples signaling pathways affected by RSV infection, an integrative model based on systems pharmacology predictions has been used. Moreover, this methodology will allow understanding the effect of flavonoid (FLA) compounds against RSV infection, integrating chemical-protein (CP) and protein-protein interaction (PPI) networks.

## 2. Materials and Methods

### 2.1. Gene Expression Data from Primary Human Bronchial Epithelial (PHBE) Cells Infected by RSV

The microarray data GSE12144 were downloaded from the Gene Expression Omnibus (GEO) database [http://www.ncbi.nlm.nih.gov/geo/]. Subsequently, a linear model was applied to normalize this data, using Limma package from R/Bioconductor to guarantee maximal statistical stringency [17]. Additionally, a contrast analysis was applied and differentially expressed genes (PHBE mock versus PHBE RSV 24h) were identified by Rank Product with a cutoff *P* value of ≤ 0.05 [18].

### 2.2. Selection of Flavonoids

To select flavonoids with potential antiviral effect against pathogenic respiratory agents, a literature mining was performed. Two flavonoids commonly described against respiratory viral infections were selected: quercetin [19–21] and resveratrol [22–24]. Quercetin is found in abundance in onions, apples, broccoli, and berries [25], whereas resveratrol is present in grapes, berries, and peanuts [25].

In order to obtain drug-like compounds, a database-dependent model was applied to calculate the drug-likeness of all compounds similar to resveratrol or quercetin through Tanimoto coefficient (Tc) [37]:

(1) $$\begin{array}{c} \text{Tc}=\frac{\sum_{j=1}^{k}a_{j}\times b_{j}}{\left(\sum_{j=1}^{k}a_{j}^{2}+\sum_{j=1}^{k}b_{j}^{2}-\sum_{j=1}^{k}a_{j}\times b_{j}\right)}, \end{array}$$

where “*a*” is the molecular property of each compound and “*b*” represents the average molecular properties of the whole compounds in the Drugbank database [http://www.drugbank.ca/]. The Drugbank database is a unique bioinformatic resource that contains 6825 compound data. These chemical compounds are FDA approved drugs or are being evaluated in clinical trials. In our work a criterion of Tc values ≥ 0.611 was used according to suggestion by Drugbank site (data shown in the Table 1).

### 2.3. Design of CP-PPI Networks

To obtain CP-PPI networks, the metasearch engine STITCH 3.1 [http://stitch.embl.de/] was applied. STITCH software allows visualization of the connections (edge) among different proteins, chemical compounds, and compounds-proteins, where each edge shows a degree of confidence between 0 (lowest confidence) and 1.0 (highest confidence). To this present work, the parameters used were as follows: all prediction methods were enabled, excluding text mining; maximal of 10 interactions by node; degree of confidence, medium (0.400); and a network depth equal to 1. In addition, GeneCard [http://www.genecards.org/] and Pubchem [https://pubchem.ncbi.nlm.nih.gov/] databases were used to search synonymous names of genes and compounds recognizable by STITCH. In sequence, the outcomes obtained through these search engines were analyzed with Cytoscape 2.8.2 [38]. Nonconnected nodes were excluded from the networks.

### 2.4. Modular Analysis of CPI-PPI Network

ClusterONE was the tool used to discover densely connected and possibly overlapping regions within the Cytoscape network [39]. Dense regions corresponded to protein or compound-protein complexes or parts of them.

ClusterONE identifies subnetworks by the identification of “growing” dense regions out of small seeds guided by a quality function. The quality of a group was evaluated by the number of internal edges divided by the number of edges involving nodes of the group.

### 2.5. Gene Ontology Analysis

Gene ontology (GO) analysis was determined by biological network gene ontology (BiNGO) software 2.44 [http://chianti.ucsd.edu/cyto_web/plugins/index.php] [40]. The degree of functional enrichment for a given category was assessed (*P* value ≤ 0.05) by hypergeometric distribution [41] and multiple test correction was applied using the false discovery rate (FDR) algorithm [42], from BiNGO software. Overrepresented biological process categories were obtained after FDR correction, with a significance level of 0.05.

### 2.6. Centralities Parameters and Topological Analysis

Major network centralities (closeness, betweenness, and node degree) were analyzed with the CP-PPI networks using the Cytoscape plugin CentiScape 2.8.2 [43].

Closeness centrality was used to evaluate the shortest path among a random node (protein or chemical compound) and all other nodes [43]:

(2) $$\begin{array}{c} \text{Clo}\left(v\right)=\frac{1}{\sum_{}^{}w\in v^{\mathrm{dist}\left(v,w\right)}}, \end{array}$$

where the closeness value (Clo(*v*)) was calculated by computing the shortest path between the node *v* and all other nodes *w* found within a network.

The average closeness (Clo) score was calculated by the sum of different closeness scores (Clo_*i*_) divided by the total number of nodes analyzed (*N*_(*v*)_):

(3) $$\begin{array}{c} \langle \text{Clo}\rangle =\frac{\sum_{i}^{}\text{Cl}{\text{o}}_{i}}{N_{\left(v\right)}}. \end{array}$$

Also, the betweenness parameter was taken into account in the analysis. This parameter is a measure equal to the number of shortest paths from a couple of nodes that pass through a different node [43, 44]:

(4) $$\begin{array}{c} \text{Bet}\left(v\right)=\underset{s\neq v\neq w\in V}{\sum }\frac{\sigma_{sw}\left(v\right)}{\sigma_{sw}}, \end{array}$$

where *σ*_*sw*_ is the total number of the shortest paths from node *s* to node *w* and *σ*_*sw*_(*v*) is the number of those paths that pass through the node *v*.

The average betweenness score (Bet) of the network was calculated using (5), where the sum of different betweenness scores (Bet_*i*_) is divided by the total number of nodes *N*_(*v*)_ analyzed:

(5) $$\begin{array}{c} \langle \text{Bet}\rangle =\frac{\sum_{i}^{}\text{Be}{\text{t}}_{i}}{N_{\left(v\right)}}. \end{array}$$

The average betweenness score of CP-PPI network was used to obtain responsible nodes of the control of the flow of information in the network. These nodes are called bottlenecks (B) and show higher probability of connections of different modules or biological processes.

Finally, parameter degree was calculated. This parameter is a measure that indicates the number of adjacent nodes (*E*_*i*_) that are connected to a specific node (*v*), according to

(6) $$\begin{array}{c} \text{Deg}\left(v\right)=\sum E_{i}. \end{array}$$

The average node degree of a network (Deg) is given by (7), where the sum of different node degree scores (Deg_*i*_) is divided by the total number of nodes *N*_(*v*)_ present in the network:

(7) $$\begin{array}{c} \langle \text{Deg}\rangle =\frac{\sum_{i}\text{De}{\text{g}}_{i}}{N_{\left(v\right)}}. \end{array}$$

Nodes with a high node degree score compared to the average are called hubs (H) and are responsible for a central regulatory role in the cell.

In this work, H-B (hub-bottleneck) may correspond to central proteins or FLA compounds that are highly connected to several complexes, while nodes that belong to the NH (non-hub-B) group correspond to proteins or FLA compounds that are important. In order to obtain H-B and NH-nodes, mathematical means (threshold) generated for betweenness and degree parameters were considered.

### 2.7. Molecular Parameters for the Development of a Potential Drug

All compounds, which were chemically verified by Zinc database [45, 46] were analyzed taking into account the Lipinsky's rule of five (xLogP, molecular weight, number of hydrogen bond acceptors, and donors). Toxicity risks (mutagenic, tumorigenic, irritant, and reproductive effect) were also examined by the Osiris Property Explorer [http://www.organic-chemistry.org/prog/peo].

**Figure 1:**
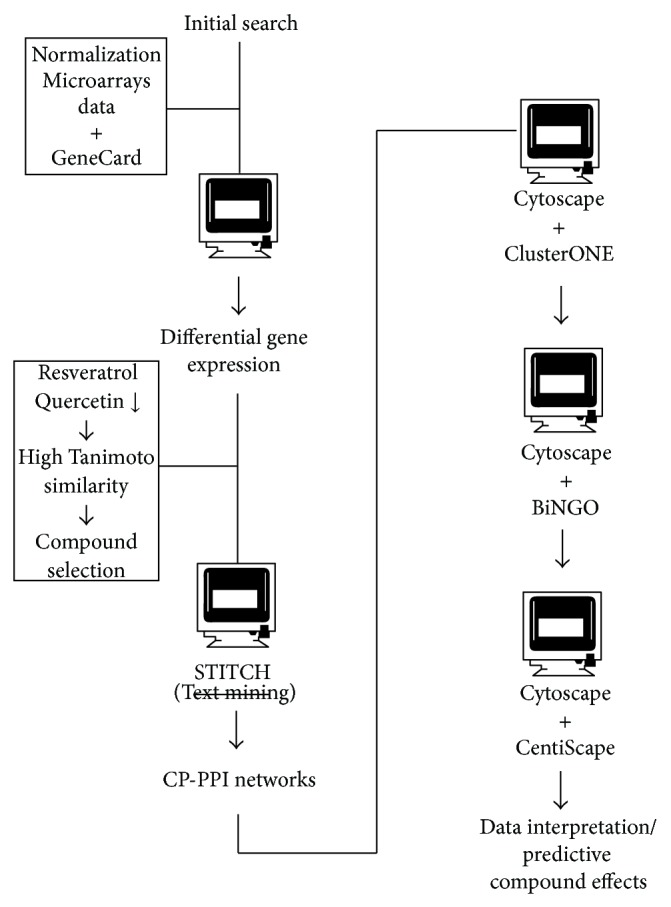
Experimental approach employed to define potential treatments against RSV infection. The interactome data was obtained from microarrays data derived from human bronchial cells infected with RSV. Differential gene expression was considered as initial input for network prospection. Additionally, the natural compounds from flavonoids obtained according to Tanimoto similarity were added to the initial input in STITCH software. The CP-PPI network generated was viewed by Cytoscape and analyzed by ClusterONE in order to identify the major clusters associated. Biological processes found within clusters were retrieved by employing BiNGO plugin. Moreover, to find bottlenecks and hubs, proteins/compounds used CentiScape plugin. Finally, data interpretation was performed based on Zinc database and Osiris Property Explorer.

A diagram of methodological steps used in this work is showed in Figure 1.

## 3. Results and Discussion

Studies of the FLA effects on viruses only have been performed* in vitro* and* in vivo* but not* in silico* using high-throughput (*omic*)* approaches* and network analysis based on interactome data. This may occur due to the structure of flavonoids, which generally consist of two aromatic rings, each containing at least one hydroxyl group that is connected through a three-carbon “bridge” becoming later part of a heterocyclic ring [47]. These chemical proprieties allow increased permeability across the cellular membrane to interact with multiple intracellular targets [48, 49]. As such, these compounds possess a broad spectrum of biological activities [50, 51], leading to the overrepresentation of many biological pathways, which may not be necessarily linked to antiviral potential. In this sense, systems pharmacology or chemobiology strategies could be employed to define specific targets of flavonoids.

### 3.1. Topological Design and Analysis of a Major CP-PPI Network of PHBE Cells Infected by RSV

**Figure 2:**
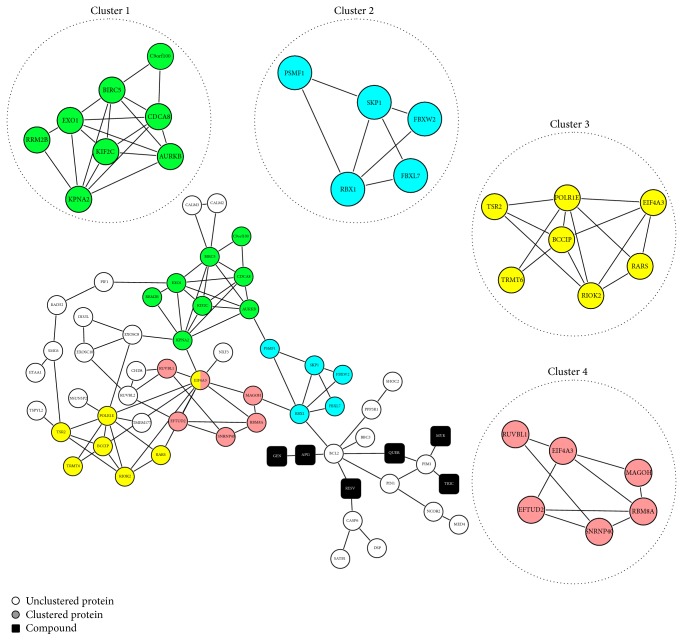
ClusterONE analysis of major chemical-protein (CP) and protein-protein interacting (PPI) network. All clustered proteins (composing different subnetworks) and unclustered proteins are represented by nodes of different colors. Chemical compounds are represented by square shape nodes. FLA compounds abbreviations: resveratrol (RESV), apigenin (APG), quercetin (QUER), myricetin (MYR), tricetin (TRIC), and genistein (GEN).

To focus on RSV antiviral effects of flavonoids, we developed an interatomic network considering 285 genes differentially expressed during RSV infection of PHBE cells and 26 flavonoids compounds (Table 1) as an initial input on STITCH software. As a result of this approach, a major CP-PPI network composed of 57 nodes and 92 edges and integrated by five compound targets with putative antiviral activity was obtained (Figure 2). It is important to note that minor networks without CPI were also detected but were not considered for posterior analysis (Supplementary Figure 1; see Supplementary Material available online at http://dx.doi.org/10.1155/2014/301635).

**Figure 3:**
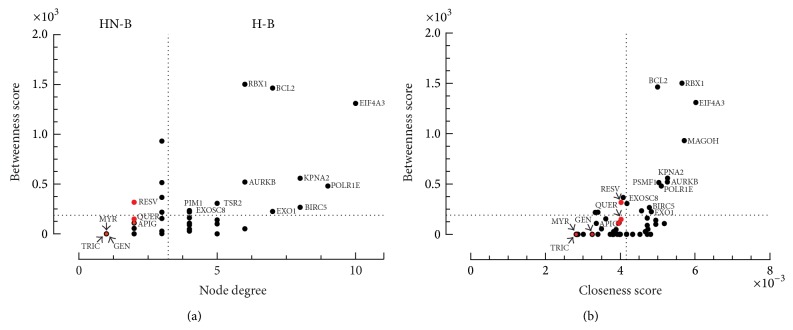
Centrality analysis (a and b) of the major CP-PPI network. Dashed lines represent the threshold value calculated for each centrality. Proteins are represented by black dots, while flavonoid compounds are marked in red. Only proteins or flavonoid compounds with bottleneck scores above the network average are indicated. Legend: hub-bottleneck (H-B); non-hub-bottleneck (NH-B). FLA Compounds abbreviations: resveratrol (RESV); apigenin (APG); quercetin (QUER); myricetin (MYR); tricetin (TRIC), and genistein (GEN).

Network topological features could successfully predict FLA mechanisms of action against RSV infection. In this sense, the global organization of clustering in the major network suitable for flavonoid modulation was analyzed. ClusterONE identified four interconnected clusters (Figure 2). Subnetworks of these clusters were created, representing four discrete biological processes, as identified by gene ontology analysis (GO) (Supplementary Table 1): (1) cell cycle phase (corrected *P* value: 2.33 × 10^−6^); (2) ubiquitin-dependent protein catabolic process (corrected *P* value: 1.61 × 10^−5^); (3) nucleic acid metabolic process (corrected *P* value: 4.68 × 10^−4^); and (4) RNA splicing (corrected *P* value: 1.65 × 10^−6^). RSV-host studies have identified these processes that occur upon infection [52–54]. However, all flavonoids and their targets are unclustered in the major CP-PPI network. This shows a compound-target regulation independent of cluster network organization during early RSV infection. An alternative and possible strategy to understand RVS modulation by flavonoids is to predict the best ranking of compound target (high impact on the network) through network connectivity analysis. In this sense, centrality properties were evaluated; however, 11 H-B nodes were identified in the CP-PPI network, represented only by proteins (Figure 3(a), Supplementary Table 2). These same H-B nodes possess high closeness values (Figure 3(b), Supplementary Table 2), suggesting that these nodes may have close communication with others in the major network. All flavonoid compounds are H-NB and NH-NB nodes, but these modulate directly 2 H-B proteins (PIM1 and BCL2).

#### 3.1.1. PIM1 and BCL2, as FLA Targets against RSV Infection

PIM1 is a protooncogene which encodes a serine/threonine kinase [55]. This kinase controls cell survival, proliferation, differentiation, and apoptosis [56]. In the context of respiratory diseases, a recent study suggests that PIM1 has a role in the induction of allergic airway responses [57]. Therefore, PIM1 inhibition reduces the development of full spectrum allergen-induced lung inflammatory responses, at least partially through limiting the expansion and actions of CD4+ and CD8 + effector T cells [57]. A similar function for PIM1 has been described in acute RSV infections [58]. PIM1 inhibition attenuates induced RSV reinfection, enhancing airway hyperresponsiveness and activation of the inflammatory cascade. In our analyses, PIM1 showed to be upregulated in comparison with noninfected control (log FC = 0.026) and to interact with three flavonoids (tricetin, myricetin, and quercetin). These compounds are cell-permeable and directly inhibit PIM1 kinase activity [59]. In this sense, these flavonoids are potential inhibitors of RSV-caused inflammation in a target-specific manner, through yet unknown mechanisms. It is important to note that anti-RSV activity of myricetin and tricetin were not tested experimentally and should be further investigated.

On the other hand, our data suggest BLC2 regulation mediated by flavonoids. BCL2 is a regulator of programmed cell death (apoptosis), in part by modulating the release of proapoptotic molecules from mitochondria. For viruses in general (included RSV), apoptotic death of infected cells is a mechanism for reducing virus replication. After 24 h of infection by RSV, several proapoptotic factors of the BCL2 family and caspases 3, 6, 7, 8, 9, and 10 are induced in different epithelial cell lines (primary small airway cells, primary tracheal-bronchial cells, A549, and HEp-2 but not for PHBE) [60]. At the same time, RSV also mediates induction of antiapoptotic factors of the BCL2 family [60], which might account for the delayed induction of apoptosis of RSV-infected cells. This indicates the importance of a complex struggle between apoptotic (host) and antiapoptotic (virus) pathways [60].

In our study, BCL2 was shown to be downregulated in PHBE infected cells in comparison with noninfected controls (log FC = −0.008). We hypothesized that differential expression of this gene may be caused by overexpression of PIM1. In hematopoietic cells, PIM1 kinase acts as a survival factor in cooperation with a regulation of BCL2 [61]. This mechanism should be investigated in RSV infected PHBE.

Furthermore, resveratrol and apigenin control the activity of BCL2 in inducing apoptosis in cancer cells [62, 63], but the effect of these flavonoids has not been explored in PHBE cells or in* in vivo* models for RSV infection. However, these compounds are described as inhibitors of RSV replication* in vitro* (see Table 1).

### 3.2. *In Silico* Analysis of FLA Effects on Human Health

We have also predicted potential undesired effects on human health of each of the FLA compounds based on its chemical structures (for more details, see Section 2.7 of Materials and Methods). Our analysis suggests that tricetin may have low risk to human health considering the four main parameters of the analysis (mutagenic, tumorigenic, irritant, and reproductive effectiveness), as shown in Table 2. The other four flavonoids (resveratrol, quercetin, apigenin, and myricetin) may require chemical modification to reduce human health impact but provide versatile chemical backbones for drug development. Biotransformation of flavonoids into drugs is the usual approach in the development of anticancer targets [64, 65] but could also be applied in the search of new therapies against RSV.

## 4. Conclusions

Our model network CPI-PPI identified five target flavonoid compounds: resveratrol, quercetin, tricetin, apigenin, and myricetin. These compounds are suggested as potential candidates in the process of development of novel drugs against early severe RSV infection. Despite these potentially interesting associations, these findings are mainly relying on statistical analysis. Thus, Further experimental testing of these predictions will be required to support the* in silico* data.

## Supplementary Material

Supplementary figure 1: All PPI networks obtained in this work. PPI network was obtained from microarrays data of PHBE cells infected with RSV, and flavonoid compounds, as initial input of STITCH software.
Supplementary Table 1: All gene ontologies obtanied from major PPI network through.
Supplementary Table 2: CentiScape Analysis. (a) Degree, betweeness and closeness centrality scores of each node in RSV CP-PPI network. (b) Hub (H) and bottlenecks (B) nodes are showed with their closeness values.

## Figures and Tables

**Table 1:** List of flavonoid compounds considered to chemical protein-protein network design. Chemical identification (Pubchem), Tanimoto similarity scores, and the antiviral activity of each compound (manually curated from literature).

Compound ID	Pubchem CID	Tanimoto similarity (score)	Antiviral RSV references
**A** ^*^			
**Resveratrol**	445154	1	[22, 24, 26–29]
Piceatannol	667639	0.966	UD^***^
AC1O4D7M	6365297	0.719	UD
Caffeic acid	689043	0.689	UD
Phenol	996	0.687	[30, 31]
HLF	5288545	0.684	UD
Sinapinate	637775	0.635	UD
Ferulic acid	445858	0.622	[32]
Isoferulic acid	736186	0.614	[32]
2MP	7249	0.621	UD
P-coumaric acid	637542	0.611	UD
**B** ^**^			
**Quercetin**	5280343	1	[19, 25]
Myricetin	5281672	1	UD
ST059620	5281614	0.959	UD
Kaempferol	5280863	0.946	[25]
Tricetin	5281701	0.884	UD
Apigenin	5280443	0.823	[33]
Oroxylin A	5320315	0.791	[34]
Wogonin	5281703	0.765	[34]
Flavone	10680	0.714	[35]
EMD 21388	128600	0.636	UD
*α*-Naphthoflavone	11790	0.711	[34]
*β*-Naphthoflavone	2361	0.711	[35]
Rutin	5280805	0.631	UD
Genistein	5280961	0.618	[36]
DB07032	656936	0.612	UD

A^*^Group with high similarity to resveratrol.

B^**^Group with high similarity to quercetin.

UD^***^Undescribed in the literature.

**Table 2:** Prediction of effects of FLA compounds based on chemical structure.

Molecules	*x*log⁡*P* ^*^	H-bond acceptors^*^	H-bond donors^*^	MV (g/mol)^*^	Mutagenic^**^	Tumorigenic^**^	Irritant^**^	Reproductive effect^**^
Resveratrol	2.99	3	3	228.247	High-risk	Low-risk	Low-risk	High-risk
Quercetin	1.68	7	5	302.238	High-risk	Medium-risk	Low-risk	Medium-risk
Apigenin	2.46	5	3	270.24	High-risk	Medium-risk	Low-risk	High-risk
Genistein	2.27	5	3	270.24	High-risk	High-risk	Low-risk	High-risk
Myricetin	1.39	8	6	318.237	High-risk	Low-risk	Low-risk	Low-risk
Tricetin	1.68	5	7	302.238	Low-risk	Low-risk	Low-risk	Low-risk

^*^All parameters related to Lipinsky's rule of five were obtained from Zinc database.

^**^All toxicity risks were predicted by Osiris Property Explorer.
