# Supplementary figures and images for: A Network Flow Approach to Predict Protein Targets and Flavonoid Backbones to Treat Respiratory Syncytial Virus Infection
Source: Biomed Res Int. 2015 Mar 22;2015:301635. doi: 10.1155/2015/301635 (PMC4386546; doi:10.1155/2015/301635)

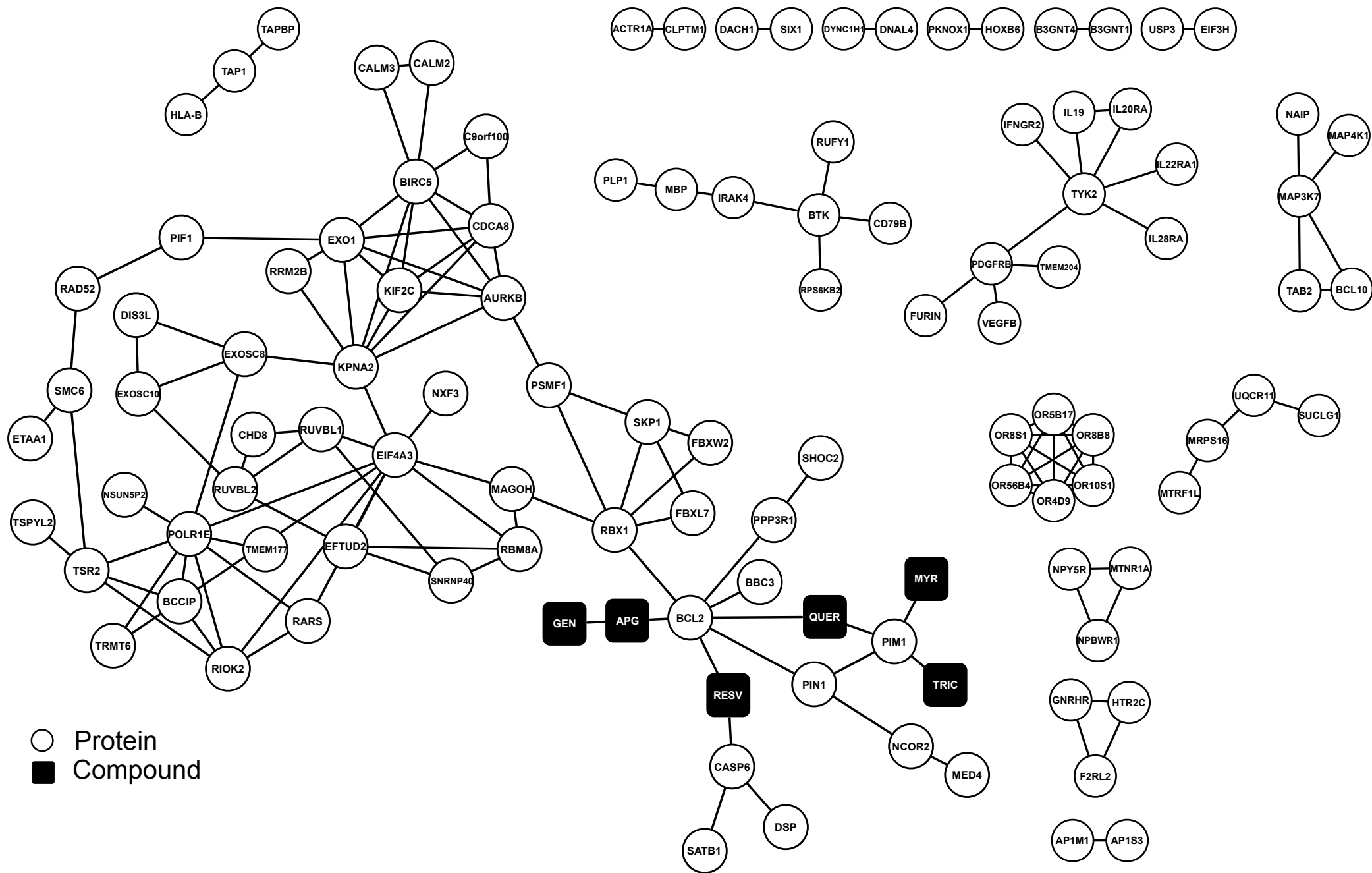

Supplement: Supplementary file 1 — Supplementary figure 1: All PPI networks obtained in this work. PPI network was obtained from microarrays data of PHBE cells infected with RSV, and flavonoid compounds, as initial input of STITCH software. Supplementary Table 1: All gene ontologies obtanied from major PPI network through. Supplementary Table 2: CentiScape Analysis. (a) Degree, betweeness and closeness centrality scores of each node in RSV CP-PPI network. (b) Hub (H) and bottlenecks (B) nodes are showed with their closeness values. [file 301635.f1.zip › Suppl Figure 1.pdf]
